# MAVS-Mediated Apoptosis and Its Inhibition by Viral Proteins

**DOI:** 10.1371/journal.pone.0005466

**Published:** 2009-03-07

**Authors:** Yu Lei, Chris B. Moore, Rachael M. Liesman, Brian P. O'Connor, Daniel T. Bergstralh, Zhijian J. Chen, Raymond J. Pickles, Jenny P.-Y. Ting

**Affiliations:** 1 Lineberger Comprehensive Cancer Center, University of North Carolina, Chapel Hill, North Carolina, United States of America; 2 Department of Oral Biology, University of North Carolina, Chapel Hill, North Carolina, United States of America; 3 Department of Surgery, University of North Carolina, Chapel Hill, North Carolina, United States of America; 4 Department of Microbiology and Immunology, University of North Carolina, Chapel Hill, North Carolina, United States of America; 5 Howard Hughes Medical Institute, Department of Molecular Biology, University of Texas Southwestern Medical Center, Dallas, Texas, United States of America; Karolinska Institutet, Sweden

## Abstract

**Background:**

Host responses to viral infection include both immune activation and programmed cell death. The mitochondrial antiviral signaling adaptor, MAVS (IPS-1, VISA or Cardif) is critical for host defenses to viral infection by inducing type-1 interferons (IFN-I), however its role in virus-induced apoptotic responses has not been elucidated.

**Principal Findings:**

We show that MAVS causes apoptosis independent of its function in initiating IFN-I production. MAVS-induced cell death requires mitochondrial localization, is caspase dependent, and displays hallmarks of apoptosis. Furthermore, MAVS^−/−^ fibroblasts are resistant to Sendai virus-induced apoptosis. A functional screen identifies the hepatitis C virus NS3/4A and the Severe Acute Respiratory Syndrome coronavirus (SARS-CoV) nonstructural protein (NSP15) as inhibitors of MAVS-induced apoptosis, possibly as a method of immune evasion.

**Significance:**

This study describes a novel role for MAVS in controlling viral infections through the induction of apoptosis, and identifies viral proteins which inhibit this host response.

## Introduction

In recent years, knowledge of host cell signaling responses to viral infection has progressed rapidly. It is known that cells of the immune system contain toll-like receptors (TLRs) capable of detecting extracellular or endosomal viral nucleic acid and activating appropriate signal transduction pathways leading to the up-regulation of immune and inflammatory cytokines. Besides detecting extracellular viral products, somatic cells can also respond to intracellular viral RNA by activating the recently identified mitochondrial antiviral signaling pathway. Following cytoplasmic detection of viral nucleic acid by the RIG-I-like helicases (RLH) family of receptors, these and other signaling proteins are recruited to the mitochondria where they interact with the mitochondrial antiviral signaling adaptor protein MAVS (IPS-1, VISA and Cardif) [1], [2], [3], [4]. *In vitro* and *In vivo* experiments have revealed a critical role for MAVS and its mitochondrial localization in the activation of host antiviral responses [1], [5]. Although the role of MAVS in type-1 interferon (IFN-I) responses is known, the localization of MAVS to the mitochondria suggests other putative mitochondrial functions for MAVS, prominent among these is apoptosis. However, to date, there are no comprehensive studies focused on testing this hypothesis. Notably, host cell apoptosis is a successful strategy to impede viral replication and restrict virus spreading during a productive infection [6].

Multicellular organisms are equipped with at least two evolutionarily conserved defensive arms to eradicate viral infections: programmed cell death and innate immune responses. Many proteins which function in both apoptotic and inflammatory signaling cascades contain a caspase recruitment domain (CARD), which functions as a homotypic interaction motif. In fact, the biological function of the CARD domain was initially described in a subset of caspases which activate mitochondria-dependent apoptotic signaling [7]. For example, the CARD containing Apaf-1 (apoptosis protease-activating factor-1) protein binds to cytochrome c and forms a ternary multimeric protein structure called the apoptosome which functions to activate caspase-9 via a proximity-induced mechanism [8]. Other CARD-containing proteins including some members of the NLR (nucleotide-binding domain and leucine-rich repeat containing) protein family have been linked with both apoptotic and inflammatory signaling [9]. For example, the CARD-containing NLR, Nod1, has been shown to activate a caspase-9 dependent apoptosis and play a positive regulatory role in pathogen-induced NF-κB activation [10]. Similarly, Nod2, a protein linked with the etiology of the autoinflammatory Crohn's disease, has been reported to augment caspase-9-induced apoptosis when overexpressed [11]. A third CARD-containing NLR, Nlrc4 (Ipaf), mediates cell death through a caspase-1 dependent fashion [12], [13], [14].

Similar to the aforementioned proteins, MAVS contains an N-terminal CARD-like domain, in addition to a central proline-rich region and a C-terminal transmembrane (TM) domain, which targets MAVS to the mitochondrial outer membrane [1]. Recent crystal structure analysis reveals that the CARD-like domain of MAVS is indeed a classical CARD fold with surface charge profiles of a typical CARD domain involved in homotypic associations [15]. Consequently, the presence of a CARD-like domain coupled with its mitochondrial localization suggests a putative role for MAVS in both immune and cell death responses. In fact, both the N-terminal CARD-like and TM domains are indispensable for MAVS-mediated activation of interferon regulatory factor-3 (IRF-3) and subsequent transcription of the antiviral IFN-I, suggesting that these domains are critical to MAVS function [1]. As a survival mechanism, it is known that some viruses have evolved strategies to inhibit MAVS function through selective targeting of these functional domains. For example, the genome of hepatitis C virus (HCV) has evolved to include a serine protease, NS3/4A, which cleaves the MAVS TM domain and dislodges MAVS from the mitochondria, thereby abrogating MAVS mediated IFN-I production [4], [16]. Similar to HCV, hepatitis A virus (HAV) encodes for the 3ABC protein, which localizes to the mitochondria and inhibits MAVS signaling via proteolytic cleavage [17], [18]. Currently, there are no reports of viral proteins targeting MAVS for inhibition of virus-induced cell death responses.

Host cell apoptosis has been reported to suppress viral replication and the subsequent production of infectious progeny viruses [19]. For example, adenoviruses and baculoviruses which are defective in anti-apoptotic genes are compromised in producing progeny viruses [19]. In addition, several viruses infectious to humans, including the coronaviruses, are known to modulate host cell apoptotic responses [20], [21]. In 2003, researchers from several labs identified a unique coronavirus linked with the pathogenesis of severe acute respiratory syndrome (SARS) in humans [22], [23], [24]. The spread of SARS-CoV, reaching near pandemic levels, resulted in the death of over 900 individuals with a case fatality rate of 11% [25]. Since that time, the sequencing of the complete SARS-CoV genome has allowed scientists to study the function of each SARS-CoV encoded protein in greater details. Phylogenetic analyses revealed that SARS-CoV was not closely related to any other characterized coronaviruses [26], [27]. The distinct nature of the SARS-CoV genome suggests possible unique strategies employed by this virus to subvert host defense mechanisms. Notably, unlike other common human respiratory viral infections, such as influenza A virus, the viral loads in the upper airway of SARS patients progressively increase, reaching a peak around 10 days after the initial onset of symptoms [28]. This indicates that at least during the initial stages of SARS-CoV infection, this virus might suppress host defensive responses. In fact, several SARS-CoV proteins have been shown to inhibit host IFN-I responses [20], [21], [29]. Although the fact that certain clinical manifestations of SARS such as lymphopenia and cell death in the lung or liver are thought to be related to the ability of SARS-CoV to induce apoptosis in specific cell types and that several pro-apoptotic proteins have been found in SARS-CoV genome [30], [31], [32], the early pathogenesis of SARS is poorly understood and how apoptosis contributes to the initial pathological changes is largely unknown. In addition, the replication of SARS-CoV seems to be restrained to the first two weeks upon symptom onset with little evidence of continued replication after this time window [33]. Clearly, the temporal and cell-type specific expression of SARS-CoV proteins could account for the dynamics of SARS-CoV infection and given that host cell fate decisions are a part of overall host responses to any pathogen, it is conceivable that like SARS-CoV inhibitory effects on IFN-I responses, this virus could employ proteins which function as inhibitors of host cell death. In this report, we describe a novel function of MAVS in mediating virus-induced apoptosis, and identify viral proteins as inhibitors of this response.

## Results

### MAVS induces caspase-dependent apoptosis

**Figure 1:**
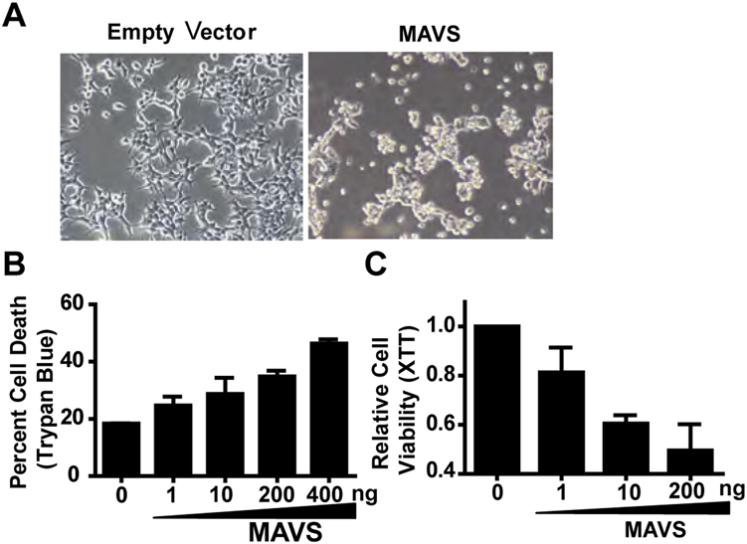
MAVS induces cell death. (A) MAVS overexpression in HEK293T cells results in cell death. (B) MAVS-induced cell death displays dose dependency determined by Trypan blue exclusion cell counting. Cells were harvested for Trypan Blue counting 48 h post-transfection. (C) In the XTT cell viability assay, 1.0×10^4^ HEK293T were plated in 96-well plate, and increasing doses of MAVS from 0 ng/well to 200 ng/well were transfected and total amount of plasmids for each well was maintained at 400 ng by mixing pcDNA3 and MAVS plasmids together. XTT assays were performed 48 hours post-transfection. Error bar represents standard deviation of triplicates of biological samples and each graph represents three individual experiments.

Transient expression of MAVS protein causes HEK293T cells to crenate and lose adherence, suggesting that MAVS is cytotoxic (Fig. 1A). Although some cell death was observed at 24 hours post-transfection, it was most evident at 48 hours post-transfection, which was quantitated by the Trypan blue exclusion test of cell viability. In this assay, we observed a dose-dependent increase in the percentage of Trypan blue positive cells at 48 hours post-transfection with MAVS plasmid (Fig. 1B). This result was confirmed by measurements of cell viability via XTT assay. MAVS-induced cell death is potent as 10 ng of MAVS plasmid was sufficient to decrease cell viability by 40% (Fig. 1C).

**Figure 2:**
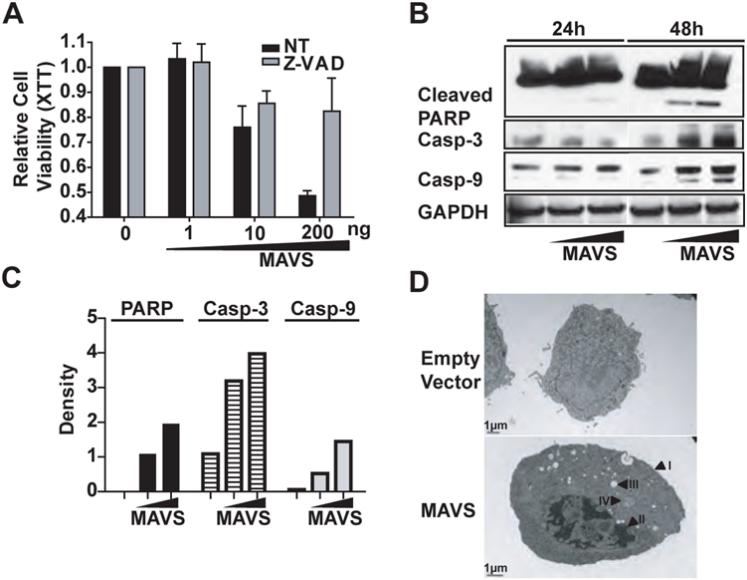
MAVS expression leads to apoptosis. (A) MAVS-induced cell death is caspase-dependent. HEK293T cells were treated with a pan-caspase inhibitor z-VAD-FMK before transfection with a titration doses of MAVS, and cell viability was measured by XTT assay. (B) MAVS triggers PARP cleavage as well as the activation of caspase-3 and caspase-9. One million HEK293T cells were transfected with 1 µg or 3 µg of MAVS-expressing plasmids and harvested 24 h or 48 h post-transfection. Cell extracts were immunoblotted for PARP, caspase-3 and caspase-9. (C) Densitometry analysis on the Western blot shown in (B). (D) Apoptotic morphological features of MAVS-induced cell death. Three micrograms of MAVS plasmids were transfected into 5×10^5^ HEK-293T cells. Transmission electron microscopy (TEM) analysis was carried out 48 hours post-transfection. Arrows: (I) intact plasma membrane; (II) marginated and condensed chromatin; (III) large vacuole; (IV) swollen mitochondria. Each graph represents two individual experiments.

MAVS contains a N-terminal CARD-like domain, which is well conserved from human to pufferfish [1]; and the CARD domain of other proteins has been shown to mediate the activation of caspases, facilitating apoptosis [34]. Therefore, we next sought to test the hypothesis that MAVS induces apoptosis through a caspase-dependent mechanism. It is well known that blockade of caspase activity will inhibit the intrinsic apoptotic pathway [35]. Thus, we treated HEK293T cells with a pan-caspase inhibitor (Z-VAD-FMK) followed by transfection of increasing amounts of MAVS plasmid. As expected, transient MAVS expression in untreated cells resulted in a significant loss of cell viability as measured by XTT assay (Fig. 2A, black bars); and application of the pan-caspase inhibitor resulted in a complete reversal of MAVS-induced cell death (Fig. 2A, grey bars). It is known that caspase-dependent apoptosis can cause Poly (ADP-ribose) Polymerase (PARP) cleavage, thus we investigated the effect of MAVS expression on the triggering of PARP cleavage. HEK293T cells were transfected with two different doses of MAVS plasmid and both adherent and floating cells were harvested and lysed 24 and 48 hours post-transfection. Immunoblotting followed by densitometry measurements revealed a dose-dependent increase in PARP cleavage (Fig. 2B, 2C). In congruence with the PARP cleavage pattern, caspases-3 and caspase-9 protein levels also showed dose-dependent increases following MAVS expression (Fig. 2B, 2C). Transmission electron microscopy examination of HEK293T cells transfected with MAVS or empty vector was performed at 48 hours post-transfection (Fig. 2D). Unlike the otherwise healthy cells transfected with empty vector (Fig. 2D, top panel), MAVS-expressing cells exhibit the morphological hallmarks of apoptosis including an intact plasma membrane (Fig. 2D, bottom panel arrow I), crenation, condensed and marginated chromatin (arrow II), large vacuoles (arrow III), cytoplasm shrinkage, membrane blebbing, an intact nuclear envelope, and swollen mitochondria (arrow IV) [36]. A kinetic analysis shows that MAVS expression reached a peak at 24 h post-transfection (Fig. S1A), while MAVS-associated apoptosis lagged behind reaching a peak at 48 h post-transfection (Fig. S1B). Thus the late onset of apoptosis does not appear to be due to the lack of MAVS expression. It is possible that there are several molecular steps that have to occur before MAVS could cause caspase-3 and caspase-9 activation, leading to apoptosis.

### Virus-induced apoptosis in primary mouse fibroblasts requires MAVS

**Figure 3:**
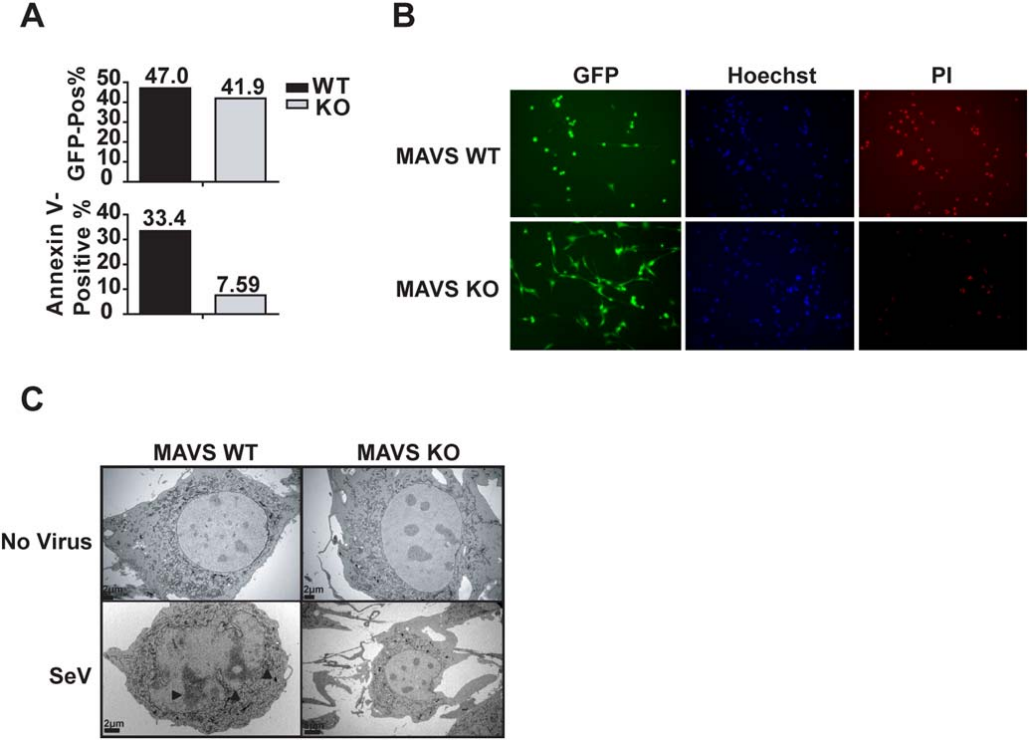
MAVS mediates SeV-induced apoptosis in primary fibroblasts. (A) MAVS is critical for SeV-induced apoptosis. MAVS wild type and knockout MEFs were infected with recombinant Sendai virus expressing GFP at a MOI of 0.5. Forty-eight hours post-infection, GFP-positive cells were gated (top graph) and then subjected to Annexin V binding analysis (bottom graph) by flow cytometry. (B) MAVS wild type and knockout MEFs were infected with rSeV-GFP at the MOI of 0.5. Forty-eight hours post-infection, cells were stained with Hoechst blue and Propidium Iodide (PI). (C) Morphological features of MAVS wild type and knock out MEFs infected by SeV. TEM pictures were taken 48 h post-infection. While SeV infected wildtype cells displayed morphology typical of apoptotic cells, SeV infected MAVS^−/−^ cells did not exhibit such morphology. Each graph represents two separate experiments.

The aforementioned studies analyzed the effect of transient expression of MAVS on cell death. To explore the physiological role of endogenous MAVS in mediating virus-induced apoptosis, we extended these findings to investigations of recombinant Sendai virus expressing GFP (rSeV-GFP) infected mouse embryonic fibroblasts (MEFs) isolated from MAVS knockout or wild type littermate control mice. MAVS deficiency was confirmed by Western blot (Fig. S2). At 48 hours post-infection, we infected MAVS^−/−^ and wildtype littermate control MEFs with rSeV-GFP, which is a known inducer of apoptosis [37]. Infection efficiency in each cell line was monitored by GFP positivity, which was similar between MAVS wildtype and knockout MEFs (Fig. 3A, top graph). In contrast, the percentage of Annexin V positive cells was >30% among infected wildtype MEFs compared to <8% for the MAVS^−/−^ MEFs (Fig. 3A, bottom graph). Similarly, MAVS-deficient MEFs maintained a spindle-like fibroblastic morphology (Fig. 3B, GFP bottom panel), while wildtype MEFs crenated and became detached from the plate. In addition, rSeV-GFP infected wildtype MEFs have higher propidium iodide/Hoechst staining ratio as compared to MAVS^−/−^, further indicating an increase in cell death (Fig. 3B, Hoechst and PI panels). Transmission electron micrographs taken for both cell types, with or without SeV, demonstrated that SeV infection resulted in the presence of typical apoptotic features in the wildtype MEFs (Fig. 3C, left panels). However, SeV infected MAVS^−/−^ showed little signs of apoptosis and are indistinguishable from the uninfected controls (Fig. 3C, right panels).

### Mitochondrial localization is required for MAVS-induced apoptosis

**Figure 4:**
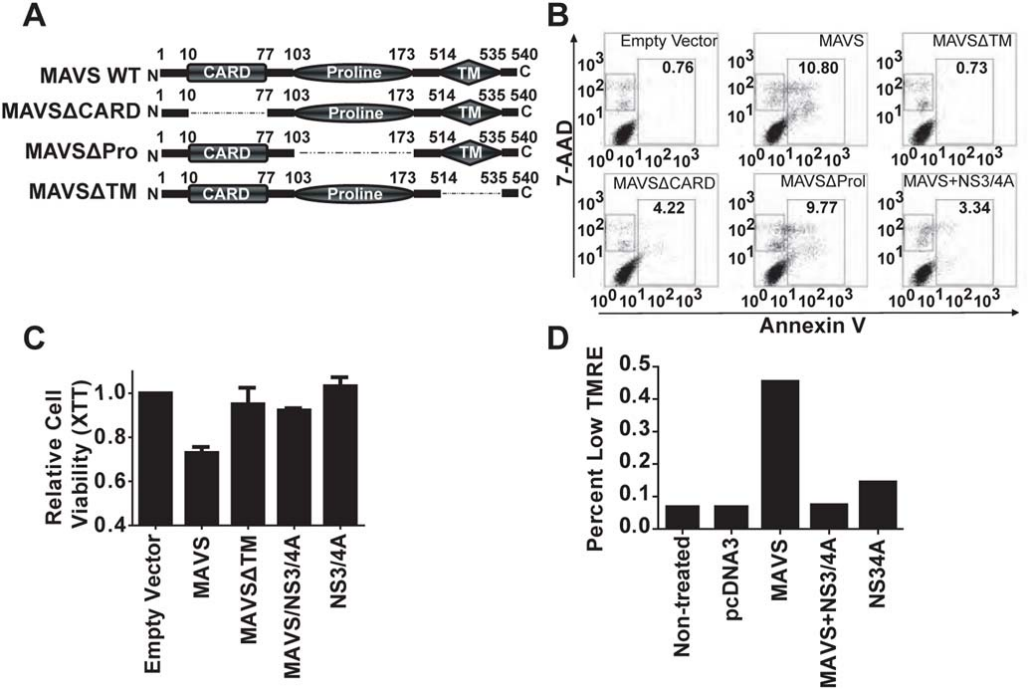
Distinct MAVS domains are required for apoptosis. (A) The domain structure of MAVS. MAVS contains a N-terminal CARD-like domain, a C-terminal transmembrane domain (TM) and a central proline-rich region. The cartoon shows the structure of the three truncation mutants we used in the study. (B) The TM and CARD-like domains are not dispensable for the pro-apoptotic function of MAVS. 1 µg of wildtype MAVS and its three mutants depicted in (A) were introduced in HEK293T cells; and apoptosis was assessed by flow cytometry 48 h post-transfection using 7-AAD and Annexin V as markers of dead and apoptotic cells respectively. (C) The mitochondrial localization of MAVS is critical for its pro-apoptotic function. Cell viability was determined by XTT assay. (D) MAVS induces mitochondrial membrane potential collapse in HEK293T cells as quantified by TMRE staining. Each graph represents two separate experiments.

Previous reports have shown that the MAVS C-terminal transmembrane domain (TM) is essential to MAVS function as an adaptor in IFN-I signaling [1]. Therefore we sought to determine the essential domain that mediates MAVS-induced apoptosis. We expressed full length MAVS protein or three truncation mutants (Fig. 4A) in HEK293T cells and measured Annexin V and 7-AAD staining. The MAVSΔTM mutant, which lacks the mitochondrial transmembrane sequence, was completely incapable of inducing apoptosis, suggesting that similar to the role of MAVS in interferon signaling, mitochondrial localization is essential to MAVS activation of apoptosis (Fig. 4B, top right panel). Truncation of the MAVS CARD domain (MAVSΔCARD) significantly inhibited MAVS-induced apoptosis, but to a lesser extent than the aforementioned MAVSΔTM mutant (Fig. 4B, bottom left panel). Truncation of the MAVS proline rich region (MAVSΔProl) had little effect on MAVS-induced apoptosis (Fig. 4B, bottom middle panel). All the MAVS truncation mutants were expressed well (Fig. S3A, S3B), thus inability of these constructs to induce apoptosis is not due to ineffective protein expression. To solidify the mitochondrial dependency of MAVS-induced cell death, we co-expressed MAVS with the hepatitis C virus (HCV) protein NS3/4A. NS3/4A is a serine protease which has been shown to target the MAVS TM domain for cleavage and subsequent inhibition of MAVS antiviral signaling [4], [17]. Consistent with its role in inhibiting MAVS mediated IFN-I signaling, NS3/4A inhibited MAVS induced apoptosis (Fig. 4B, bottom right panel). The inhibitory effects of MAVSΔTM mutant and NS3/4A were also verified by a measurement of cell viability via XTT assay (Fig. 4C). Mitochondrial membrane potential collapse marks a point-of-no-return during apoptosis and occurs earlier than DNA fragmentation [38], [39]. Only wildtype MAVS resulted in compromised mitochondrial membrane potential as measured by TMRE staining while the addition of NS3/4A abrogated this effect (Fig. 4D).

### MAVS-induced apoptosis is independent of type-1 interferon production or NF-κB activity

**Figure 5:**
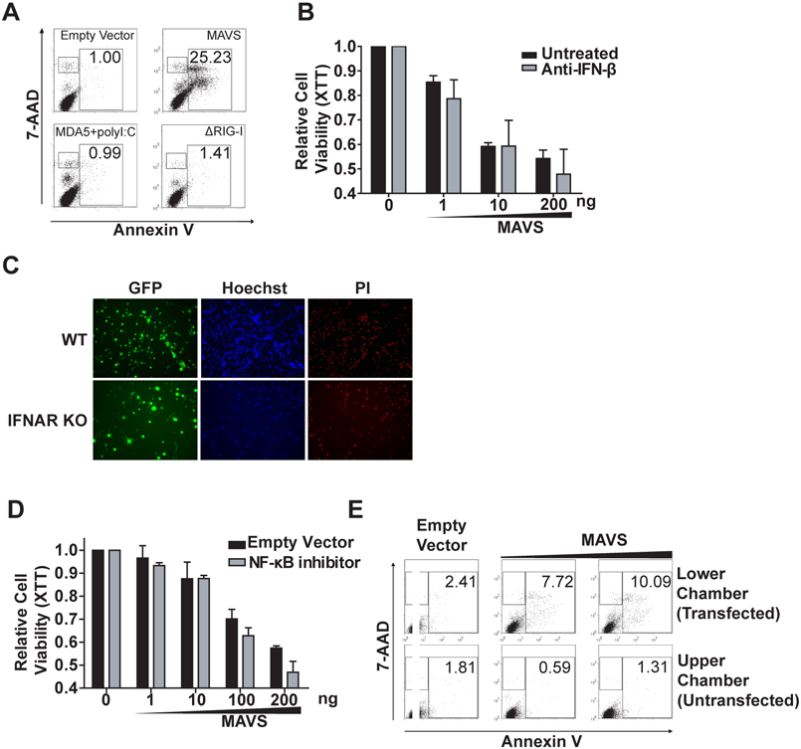
MAVS-induced apoptosis is independent of type I IFNs production. (A) Three molecules (ΔRIG-I, MDA5 and MAVS) that activate the production of type I IFNs were introduced into HEK293T cells; only MAVS is able to induce apoptosis. (B) Anti-IFN-β antibody was added to the medium at the final concentration of 200 neutralization units/ml to prevent secreted IFN-β binding to IFNAR, this neutralization process did not inhibit MAVS-induced apoptosis. (C) SeV-induced apoptosis is IFN-I-independent. MEFs from both C57BL/6 wild type and IFNAR^−/−^ mice were infected with rSeV-GFP. Cells were stained with Hoechst and PI 48 h post-infection. (D) The non-degradable form of IκB was co-expressed with different doses of MAVS, yet cell viability loss was not restored. (E) HEK293T cells were plated at both the lower chamber and upper chamber of the transwell plate separated by an insert filter membrane with 0.4 µm pore size. 1 µg of MAVS-containing plasmid was transfected into the cells in the lower chamber and all cells were harvested 48 h post-transfection for flow cytometry analysis.

**Figure 6:**
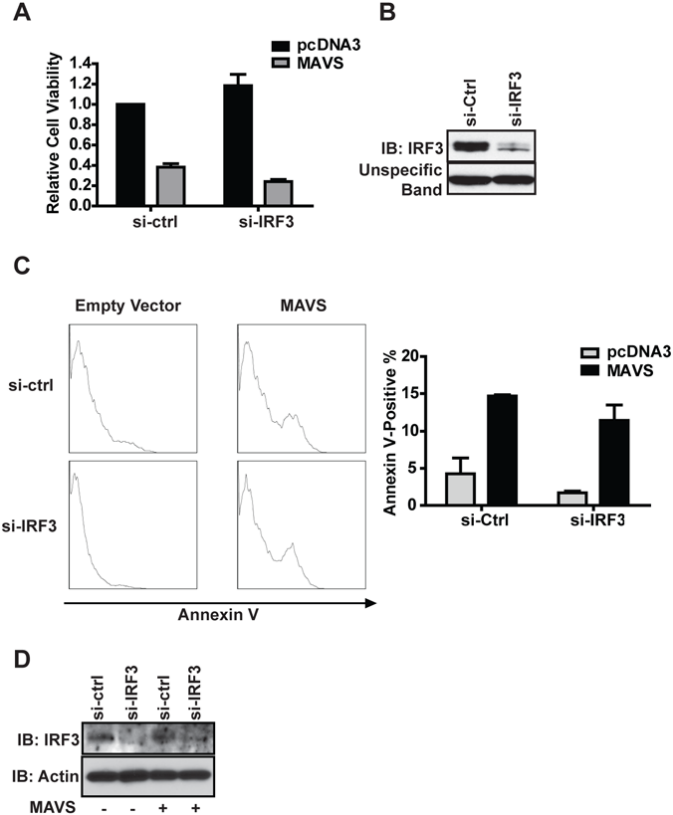
MAVS-induced apoptosis does not depend on IRF3. (A) 1.0×10^4^ HEK293T cells were plated in 96-well plates, a pool of four siRNA targeting IRF3 or control siRNA were added to each well, 24 hours later 200 ng MAVS or empty vector plasmids were transfected into the cell. XTT assay was performed 48 hours thereafter. Error bars shown in the plot represents three biological replicates. (B) The same IRF3-targeting and non-targeting siRNA transfection reagents used in (A) were applied to 5.0×10^5^ cells, and all cells were lysed in RIPA buffer 48 hours post-transfection and subjected to Western blotting to confirm knockdown. (C) 5.0×10^5^ HEK293T cells were plated in 6-well plates and treated with IRF3 or control siRNAs, 24 hours later 3 µg of MAVS expression plasmid or empty vector plasmids were introduced into the cells. Half of the cells from each well were harvested 48 hours thereafter and stained with Annexin V. The plot represents two separate experiments and the percentages of Annexin V positive cells were averaged for quantification. (D) The other half of the cells described in (C) were lysed in RIPA buffer and subjected to western blot analyses to confirm IRF3 knockdown.

It has been shown that exogenous MAVS expression triggers IFN-I production [1], [5]. Type-1 IFNs mediate their effects by binding to cell surface receptors, activating downstream interferon-stimulated genes or ISGs, among which more than 15 genes have pro-apoptotic functions [40]. We sought to determine if MAVS-induced apoptosis was the result of activation of a distinctive signaling pathway or as a consequence of IFN production. First, we induced IFN-I production in HEK293T cells by ectopic expression of upstream signaling molecules that activate MAVS-mediated IFN production. As previously reported, a helicase domain truncation mutant of RIG-I [41], full-length MAVS [1], or MDA-5 in conjunction with its ligand poly(I∶C) [42] zare all potent IFN-I inducers (Fig. S4). Neither ΔRIG-I nor MDA-5 plus poly(I∶C) induced any observable cell death or Annexin V-positive cells (Fig. 5A, bottom panels). Only full-length MAVS triggered apoptosis as quantified by Annexin V and 7-AAD staining (Fig. 5A, top right panel). To explore the role of IFN-β in MAVS-induced apoptosis, HEK293T cells were treated with 200 neutralization IU/ml IFN-β antibody prior to transient expression of MAVS followed by XTT measurements. We found that blocking IFN-β from binding to its cell surface receptor had no effect on MAVS-induced apoptosis (Fig. 5B) even though the antibody efficiently blocked the function of secreted IFN-β (Fig. S5). In congruence with these findings, rSeV-GFP infections of the interferon-α/β receptor (IFNAR) knockout and wildtype MEFs demonstrated that this receptor is not required for SeV-induced cell death as visualized by PI staining (Fig. 5C). MAVS is also capable of inducing the transcription factor NF-κB, which is known to activate a wide array of genes linked to immune, inflammatory, and cell-fate processes [43]. To test the involvement of NF-κB in MAVS-induced apoptosis, we co-expressed MAVS with the non-degradable super-repressor form of IκBα. This super-repressor is a known potent inhibitor of NF-κB activation and as expected inhibited MAVS-induced NF-κB activation (Fig. S6) [44]. However, transient expression of MAVS in HEK293T cells induced apoptosis even in the presence of the NF-κB super-repressor (Fig. 5D). There are 14 subtypes of type-1 IFNs, and a plethora of other secreted soluble factors can be induced by MAVS expression. Therefore, we performed a transwell assay designed to definitively answer if MAVS-induced cell death was intrinsic to the host cell or caused by some unknown secretory factor. HEK293T cells were plated in two different chambers (top and bottom) separated by an insert filter membrane, permissive to all secretory cytokines but impermeable to FuGENE6:DNA complexes. In this experiment, only the lower chamber was transfected with MAVS. Cells in the upper chamber were not transfected with MAVS, but were exposed to the same cytokine milieu. As expected, MAVS induced cell death in the lower chamber (transfected cells); however the cells in the upper chamber (untransfected cells) remained healthy and unchanged from controls (Fig. 5E). This indicates that the MAVS-induced death is not caused by an undefined secretory product(s) which includes the interferon family members. IRF3 is a critical transcription factor for IFN-I production but it has been associated with apoptosis, and furthermore can be activated by MAVS [37], [45]. We assessed whether MAVS-induced apoptotic signaling depends on IRF3. We made numerous attempts to introduce MAVS into IRF3^−/−^ fibroblasts, however these cells were much more difficult to transfect than wildtype fibroblasts, hence the different efficiencies of transfection made the interpretation of data difficult. Instead we used siRNA to reduce IRF3 expression. Briefly, HEK293T cells were plated in 96-well plate and endogenous IRF3 expression was reduced by transfecting a pool of four siRNA into the cells. MAVS was introduced 24 hours after siRNA transfection. Cell viability was measured by XTT assay 48 hours after cells were transfected with an expression plasmid containing MAVS or a control empty vector. The reduction of IRF3 did not prevent MAVS-induced loss of cell viability (Figure 6A). The same siRNA was introduced into 5×10^5^ cells plated in a 6-well plate, and an immunoblot was used to verify the efficiency of siRNA 48 h post-transfection (Figure 6B). As an alternate approach to measure cell death, the experiment was repeated in 6 well plates and cells were harvested 48 hours after plasmids transfection. Half of the cells from each well were stained for Annexin V and analyzed by flow cytometry, and the other half of the cells were lysed in RIPA buffer for Western blotting analyses of IRF3. As expected we were able to greatly reduce the endogenous IRF3 expression, yet MAVS-induced apoptosis was not abrogated (Fig. 6C, 6D).

### The SARS-CoV non-structural protein 15 inhibits MAVS-induced apoptosis

**Figure 7:**
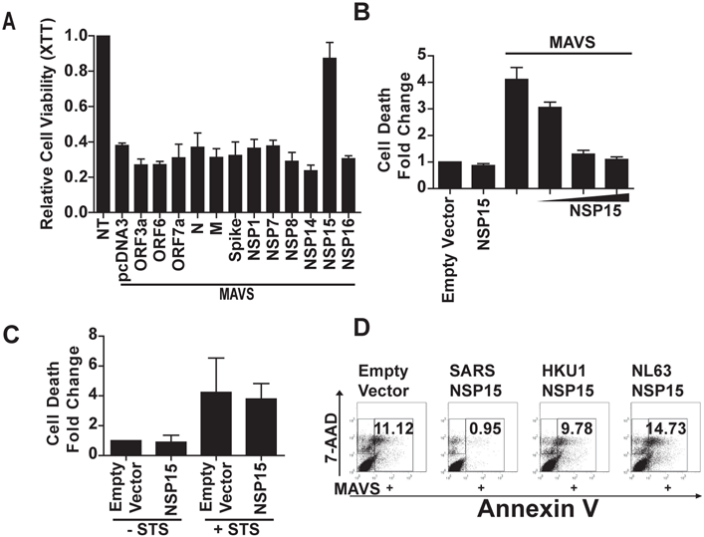
The SARS-CoV NSP15 protein abrogates MAVS-induced apoptosis. (A) Twelve of the SARS-CoV proteins were co-expressed with MAVS and NSP15 is the only one that exhibited a potent inhibitory effect on MAVS-induced apoptosis as assessed by XTT cell viability assay. (B) The inhibitory effect of NSP15 displays dose-dependency. Cell death was measured by an adenylate kinase activity assay. (C) The anti-apoptotic function of NSP15 does not extend to staurosporine-induced cell death. NSP15-expressing plasmid or empty vector were transfected in HEK293T cells seeded on 96-well plate, cells were treated with 500 nM staurosporin or PBS 24 h post-transfection, cell death fold change was evaluated by the adenylate kinase activity assay. (D) The inhibitory effects of NSP15 is unique to SARS-CoV. MAVS was co-expressed with NSP15 encoded by SARS-CoV, HKU1 and NL63. Flow cytometry analysis of 7-AAD/Annexin V stained cells was performed 48 h post-transfection. The results represent two separate experiments.

While host response that elicits apoptosis may function as an antiviral strategy, viruses have also evolved diverse mechanisms to evade these host antiviral responses. Therefore we performed a functional screen of 12 SARS-CoV-encoded proteins to identify any potential modulators of MAVS-induced apoptosis. Each of these SARS-CoV genes were cloned into an expression vector and co-expressed with MAVS plasmid followed by XTT cell viability measurements. Protein expression was verified by an immunoblot (Fig. S7). Of the 12 tested proteins, only one SARS-CoV protein, NSP15, significantly altered MAVS-induced apoptosis of HEK293T cells (Fig. 7A). Further examination showed that NSP15 significantly inhibited MAVS-induced apoptosis in a dose-dependent manner (Fig. 7B). The anti-apoptotic function of NSP15 displayed specificity since it did not inhibit staurosporine-induced apoptosis (Fig. 7C). We sought to investigate if the anti-apoptotic function of NSP15 was specific for SARS-CoV or shared by other coronaviruses. When the NSP15 protein encoded by the SARS-CoV, HKU1 or NL63 genome was coexpressed with MAVS, only SARS-CoV NSP15 inhibited MAVS-mediated apoptosis as measured by Annexin V and 7-AAD staining, while the others showed little effect on cell viability (Fig. 7D).

## Discussion

**Figure 8:**
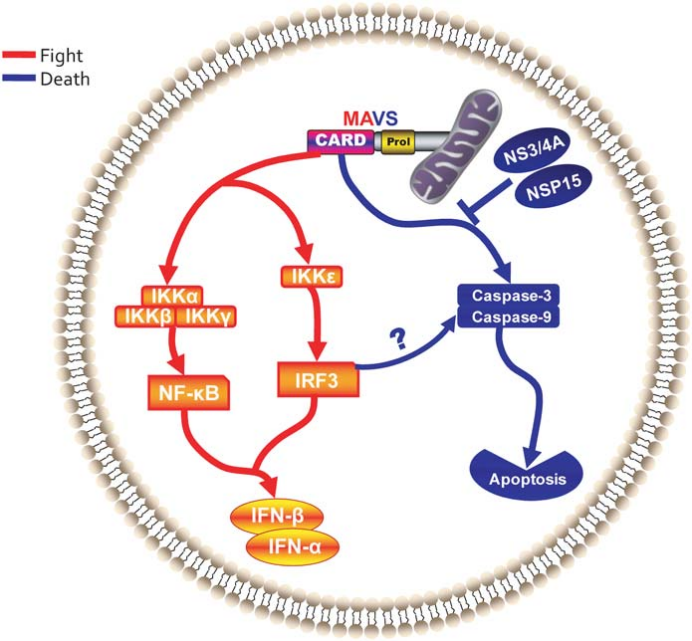
A proposed model for the dual functions of MAVS. MAVS is engaged in two distinct host protective responses to viral infections. Upon activation by RNA viruses, MAVS initiates type 1 IFN signaling by activating the nuclear translocation of NF-κB and IRF3. In addition, host cells could mount apoptotic responses to viruses such as SeV via MAVS. Notably MAVS is targeted by viruses not only for abrogating IFN-I production but also inhibiting host apoptosis. For example, HCV NS3/4A and SARS-CoV NSP15 proteins abrogate MAVS-mediated apoptosis.

Host cellular response to virus infection involves the concomitant activation of parallel signaling pathways leading to the transcription of a plethora of cytokine genes, prominent among these are the genes encoding type-1 interferons (IFN-I). It is the autocrine and paracrine action of these and other cytokines which encompasses the comprehensive host immune response designed to defend against viral infection. This is accompanied by a reciprocal activation of programmed cell death in infected host cells, which is also known to reduce viral load. Apoptosis has been suggested to play a protective role at the organismal level in preventing the virus from completing its replication and producing infectious progeny viruses [19]. Consequently, the exact mechanisms underlying both virus-induced apoptotic signaling in addition to viral strategies to subvert these responses is currently a topic of intensive research. While pathways that govern IFN-I have been extensively investigated, the molecular mediators that activate host cell apoptosis during viral infection are less known. In this study we have identified a role for MAVS in the initiation of virus-induced apoptosis (Fig. 8). Furthermore, we have identified hepatitis C virus NS3/4A and SARS-CoV NSP15 proteins as inhibitors of MAVS-mediated apoptosis.

It is known that MAVS is a potent inducer of IFN-I responses and that IFN-I can activate host apoptotic responses, therefore it was important to determine whether the cell death responses observed in the current study were a consequence of IFN-I secretion. In fact, IFN-I consist of several species including IFN-α, IFN-β, IFN-ω and IFN-κ and these cytokines bind to surface receptors leading to the activation of the Jak-Stat signal transduction pathway resulting in the transcriptional activation of virtually hundreds of IFN-stumulated genes (ISGs), whose protein products play pivotal roles in a variety of biological events, including but not limited to immunomodulation, cell differentiation, anti-angiogenesis and programmed cell death. For example, type-1 IFN induction of apoptotic responses can be mediated by a multitude of ISGs, illustrated by the finding that more than 15 ISGs have pro-apoptotic functions [40]. In addition, the involvement of proteins on IFN axis in virus-induced host cell apoptosis has been implicated in another previous report, in which MAVS has been shown to be critical for reovirus-triggered caspase-3/7 activation in HEK293T cells [46], however, the study did not evaluate whether MAVS mediates virus-induced apoptosis and what roles type 1 IFNs play in MAVS-mediated apoptosis. Using a multi-pronged approach we demonstrate that MAVS-mediated apoptosis is not a consequence of IFN-I induction by MAVS. We show that (a) the over-expression of the truncation mutant of RIG-I that induce IFN-I does not lead to apoptosis; (b) anti-IFN-β antibody does not ablate MAVS-induced apoptosis; (c) the targeted deletion of IFN receptor does not alter MAVS-induced apoptosis; (d) the co-culture of MAVS-transfected cells with nontransfected cells separated by an insert filter membrane does not induce apoptosis in the latter, indicating that a soluble secretory factor is not likely responsible for the apoptosis-inducing activity.

In the IFN-I induction pathway mediated by endogenous RIG-I like helicases, IRF3 lies downstream of MAVS, and exogenous expression of MAVS can lead to the phosphorylation and nuclear translocation of IRF3 [1]. In addition, others have shown that the expression of constitutively active form of IRF3 mutant is toxic to cells and the transfection of wild type IRF3 expression can augment SeV-induced apoptosis [37], [45]. Hence it was important to evaluate if MAVS-induced apoptosis depends on the presence of IRF3. Our data shows that depletion of endogenous IRF3 by means of RNAi did not reverse MAVS-induced cell viability loss. Together with our findings that MAVS-induced apoptosis is IFN-I-independent, we speculate that MAVS-induced apoptotic signaling pathway is different from the classical MAVS-mediated IFN-I response. Furthermore, it is possible that the reported pro-apoptotic effects of the constitutively active form of IRF3 might be independent of its IFN-I-inducing function. In fact our data suggests other IFN-I signaling molecules such as the constitutively active form of RIG-I and MDA5 do not lead to apoptosis despite of their roles in inducing IFN-I.

The dual functions of MAVS in virus-induced IFN-I and apoptosis highlight this molecule as a putative target for viral evasion strategies designed to escape host immunity. For example, it is already known that Hepatitis C Virus (HCV) produces a serine protease, NS3/4A, which disrupts IFN-I production through the targeted cleavage of the MAVS transmembrane region and the subsequent dislodging of MAVS from the mitochondria [4], [16]. In fact, we found that loss of MAVS mitochondrial localization through mutation of the transmembrane domain, also completely abolished its pro-apoptotic effects. Consistent with these results, when HCV NS3/4A protein was co-expressed with MAVS, this viral protein completely inhibited MAVS-induced cell death. This result would suggest that the host immune evasion strategies of the HCV NS3/4A protein may be extended to inhibition of MAVS-mediated apoptosis. Similarly hepatitis A virus (HAV), a picornavirus, employs a cysteine protease 3ABC to abrogate IFN-I production by targeting MAVS [17]. Therefore, it is likely that other uncharacterized viral mechanisms target MAVS for modulation of host cell death responses.

One novel strategy described in this study is employed by the SARS-CoV. Similar to SARS-CoV abilities to inhibit IFN-I responses, we have found that this virus is also capable of interfering with host cell death responses by targeting MAVS. We showed that among the 12 SARS-CoV proteins tested, NSP15 alone can completely abrogate MAVS-induced apoptosis. The underlying mechanism is currently unclear since the function of NSP15 is not yet fully defined. However, an earlier report indicates that this protein is indeed important for SARS pathogenesis in that it is essential for viral replication [47]. However, until we understand the exact temporal expression patterns of each of the SARS-CoV proteins during the course of an infection, the relative contributions of each to the evasion of host immunity and apoptosis will not be fully understood. Further studies are needed to determine the exact mechanism of action for NSP15 on inhibiting MAVS-induced cell death. Since SARS-CoV replication is limited to the first two weeks after symptom onset and NSP15 is critical for its replication [33], [47], this would support the theory that NSP15 may function as an inhibitor of host cell death during this time, which would possibly benefit viral replication. Further studies are ongoing to determine how NSP15 and other SARS-CoV proteins contribute to overall viral evasion strategies.

The finding that MAVS mediates virus-induced apoptosis posits a new mechanism by which mitochondria serves as a *bona fide* intracellular sentinel for antiviral and apoptotic responses. It is well established that the permeabilization of the mitochondria outer membrane by the pro-apoptotic Bcl-2 family member BAK results in the activation of caspase-9 in a classical Apaf-1-dependent or an alternate Apaf-1-independent pattern [48]. Furthermore, it is known that proteins localized on the mitochondria are targeted by viruses to modulate host responses. For example, the cytomegalovirus RNA can interact with the mitochondrial enzyme complex I (reduced nicotinamide adenine dinucleotide–ubiquinone oxido-reductase) to modulate the classical mitochondria-mediated apoptosis [49]. Recent discoveries also underscore the mitochondria as a platform orchestrating host antiviral type-1 IFNs through the mitochondrial MAVS protein. This study describes, for the first time, a duality of function for the MAVS protein in regulating both IFN-I and apoptotic antiviral responses from within the mitochondria and suggests that MAVS is a pivotal molecule in the bifurcation of host responses following viral challenge. Furthermore, the identification of HCV NS3/4A and SARS-CoV NSP15 as inhibitors of MAVS-mediated apoptotic responses reveals both novel host defense mechanisms as well as viral immune-evasion mechanisms that might serve as useful templates for the development of anti-viral drug strategies.

## Materials and Methods

### Cells and Plasmids

HEK293T cells, MAVS^+/+^, MAVS^−/−^, IFNAR^−/−^ mouse embryonic fibroblasts were maintained in DMEM media supplemented with 10% FBS, 1% penicillin and 100 µg/ml streptomycin. Cells were passed every three days and grown at 37°C in 5% CO_2_.

The mammalian expression plasmids of NS3/4A, wild type MAVS and truncation mutants were kindly provided by Dr. Zhijian Chen at the University of Texas southwestern medical center. HA-tagged SARS-CoV proteins expression plasmids, HKU1 NSP15 and NL63 NSP15 expression plasmids were provided by Drs. Ralph Baric and Matthew Frieman at the University of North Carolina.

### Transfections and Viral Infections

HEK293T cells were seeded in 96-well or 6-well plates, and the total DNA transfected into these cells was 400 ng/well and 1 µg/well respectively. Standard transfection protocol was performed using FuGENE6 (Roche Applied Science) according to the commercial protocol. Cells were incubated for the indicated times prior to assay.

rSeV-GFP is a recombinant Sendai virus expressing GFP and was originally made by Dr. Daniel Kolakofsky [50]. For viral infections, 1.6×10^4^ MEFs were plated into 12-well plate one day prior to Sendai virus (SeV) infection. Viral infections were performed when cells reached 60% confluence. MEFs were incubated with SeV for 1 h in serum-free DMEM supplemented with 0.5% trypLE Select at the MOI of 0.5. Serum-free DMEM supplemented with 0.5% trypLE Select was used for mock infection. Virus inoculum was removed and cells were replenished with complete media supplemented with 0.5% trypLE Select following incubation with virus.

### Cell Viability Assays

XTT salt (Sigma, St. Louis, MO) (1 mg/ml) was dissolved in serum-free media and phenazine methosulfate (PMS) (5 mM) (Sigma, St. Louis, MO) solution was prepared fresh. For activated XTT solution, both solutions were mixed at a ratio of 5 µl of PMS per 1 ml XTT. 50 µl of activated XTT solution was added to each 96 well (1×10^4^ cells/well), incubated at 37°C for 4 hours and absorbance was read at a wavelength of 450 nm. An adenylate kinase non-destructive cytotoxicity assay was performed utilizing the ToxiLight bioassay kit (Lonza) following the manufacturers standard protocol for adherent cells in a 96 well plate. For Trypan blue exclusion test of cell viability, 10 µl of cell suspension was mixed with 10 µl of 0.4% Trypan blue solution (Sigma, St.Louis, MO), and both unstained (viable) and stained (dead) cells were counted on the hemacytometer. The percent of dead cells was calculated by dividing the number of stained cells by the total number of cells.

### Flow Cytometry

Cells were harvested and washed in cold FACS buffer (5% FBS in 1× PBS) twice and in 1× Annexin V binding buffer (BD Bioscience, San Diego, CA) once. Cells were transferred into 96-well plate and resuspended in 45 µl Annexin V binding buffer. Cells were stained according to standard cell staining protocol [51] using Annexin V conjugated to FITC or APC (BD Biosciences, San Diego, CA). Cells were washed in FACS buffer and stained with 7-AAD (Invitrogen, Carlsbad, CA) for 10 min at room temperature. Cells were washed twice and resuspended in FACS buffer containing 1 µg/ml Actinomycin D. After 5 min incubation, all samples were fixed in 500 µl 1% EM grade formaldehyde (Polysciences, Warrington, PA) for immediate flow cytometry analyses. For the rSeV-GFP infected samples, GFP positive cells were gated; Annexin V and 7-AAD positive populations were assessed thereafter.

Mitochondrial membrane potential Δψ was assessed by flow cytometry analysis on a Δψ- sensitive fluorophore tetramethylrhodamine ethylester (TMRE) (Molecular Probes, Eugene, OR). After treatment, HEK293T cells were incubated with TMRE at the concentration of 10 nM per 1×10^6^ cells for 40 min at 37°C. Immediate flow cytometry analysis was performed after staining.

All flow cytometry data were collected on either a FACSCalibur (BD Biosciences, San Jose, CA) or CyAn flow cytometer (Dako North America, Carpinteria, CA) and then analyzed by FlowJo software (Tree Star, Ashland, OR).

### Imaging

Transmission electron microscopy examination was performed as described [52]. MEFs or HEK293T cells were harvested and fixed in glutaraldehyde and all samples were post-fixed in 1% OsO_4_ for transmission electron microscopy examination. Cells were embedded in London Resin White and sections were analyzed using a LEO EM-910 transmission electron microscope (LEO Electron Microscopy Inc., Thornwood, NY). Hoechst and Propidium Iodide (PI) dual staining was used to evaluate SeV-induced cell death. Samples were photographed using a Leica DMIRB inverted fluorescence microscope (Leica Microsystems Inc., Bannockburn, IL) with a digital camera (MicroPublisher, Q-Imaging, Burnaby, BC, Canada).

### Immunoblotting

Cells were lysed in RIPA lysis buffer (1% Triton X-100, 0.25% DOC, 0.05% SDS, 50 mM Tris pH8.0, 150 mM NaCl and 50 mM NaF) containing proteinase inhibitor cocktails (Roche) for 30 min at 4°C. Whole cell lysates were suspended in Laemmli's sample buffer and loaded onto NuPAGE Bis-Tris 4–12% gradient pre-cast gels (Invitrogen, Carlsbad, CA) for SDS-PAGE and subsequent immunoblotting for PARP, caspase-3 and caspase-9 (Abcam, Cambridge, MA), IRF3 (Santa Cruz Biotechnology, Santa Cruz, CA), rodent specific MAVS (Cell Signaling, Danvers, MA). Densitometry was performed using ImageJ analysis software.

### Caspase and Interferon inhibition treatments

HEK293T cells were incubated with 50 µM Z-VAD-FMK to inhibit caspase activity. HEK293T cells were treated with human IFN-β neutralizing antibody (GeneTex, San Antonio, TX) at the concentration of 200 neutralization units/ml.

### Small interference RNA depletion of endogenous IRF3

HEK293T cells were transfected with a pool of four targeting siRNA according to the manufacturer's protocol (Dharmacon, Chicago, IL). The sequences are: (1) sense 5′-CGAGGCCACUGGUGCAUAUUU, antisense 5′-AUAUGCACCAGUGGCCUCGUU; (2) sense 5′-CCAGACACCUCUCCGGACAUU, antisense 5′-UGUCCGGAGAGGUGUCUGGUU; (3) sense 5′-GGAGUGAUGAGCUACGUGAUU, antisense 5′-UCACGUAGCUCAUCACUCCUU; (4) sense 5′-AGACAUUCUGGAUGAGUUAUU, antisense 5′-UAACUCAUCCAGAAUGUCUUU. A pool of 4 non-targeting siRNAs were used as control. Empty vector or MAVS expression plasmids were introduced into the cells 24 hours after the transfection of siRNA.

### Transwell Assay

HEK293T cells were seeded on the bottom of both chambers in a HTS 24-well transwell plate with 0.4 µm pore polycarbonate membrane (Corning Incorporated Life Sciences, Lowell, MA). MAVS expression plasmid DNA was transfected into the cells on the bottom of lower chamber and flow cytometry analysis on apoptosis was performed 48 h post-transfection on cells grown in each chamber.

## Supporting Information

Figure S1 The kinetics of MAVS expression and MAVS-induced apoptosis. (A) 5×105 HEK293T cells were plated in 6 well plate, 3 µg MAVS plasmids were transfected at 3 h, 12 h, 24 h and 48 h prior to cell harvesting. Half of the cells were lysed in RIPA buffer and blotted with anti-FLAG to determine the protein expression kinetics. (B) The other half of the cells from each well were stained with Annexin V and analyzed by flow cytometry.
(11.68 MB TIF)

Figure S2 Confirmation of MAVS knockout by Western Blot. One million MAVS+/+ and MAVS−/− MEFs were lysed in RIPA buffer containing proteinase inhibitor cocktail. Cell extracts were subjected to SDS-PAGE and Western blotting for rodent-specific MAVS.
(2.15 MB TIF)

Figure S3 Expression test of MAVS truncation mutants. 3 µg FLAG-MAVS wild type, FLAG-MAVSΔCARD, FLAG-MAVSΔpro and HA-MAVSΔTM plasmids were transfected to 5×105 HEK-293T cells seeded in 6 well plate. Cell were harvested 24 h post-transfection and blotted with anti-FLAG or anti-HA antibody to confirm protein expression efficiency.
(4.71 MB TIF)

Figure S4 MAVS, ΔRIG-I and MDA5 plus Poly (I∶C) induce increased transcription of IFNB1 and IFNA4. 5×105 HEK293T cells were seeded in 6-well plates and grown overnight. 1 µg of MAVS and ΔRIG-I plasmids were transfected into the cells the next day, similarly 1 µg MDA5 and 100 ng poly (I∶C) were co-transfected into the cells as well. RNA samples were extracted from each group of cells 24 h post-transfection and subjected to real time RT-PCR analyses on the transcripts of IFNB1 and IFNA4.
(5.59 MB TIF)

Figure S5 IFN-β neutralizing antibody is able to block the function of secreted IFN-β. A549 cells were seeded in 96-well plate at the density of 2×104 per well, 1 µl of media or anti-IFN-β antibody was added to reach the final concentration of 200 neutralization IU/ml the next day. One hour after incubation with the antibody, recombinant IFN-β was added to culture at a series of doses from 31.25 to 500 IU/ml. Cells were infected with Encephalomyocarditis virus (EMCV) at 4×106 pfu/ml 16 h after IFN treatment. The plates were blind scored 24 h post-infection using “1” as maximal protection (most cells are protected by IFN), “0.5” as about 50% of the cells were not protected by IFN, and “0” as no IFN protection (most cells are dead).
(6.29 MB TIF)

Figure S6 NF-κB super-repressor inhibits the activity of NF-κB. HEK293T cells were seeded in 96-well plate at the density of 1×104 per well and grown overnight. 25 ng of NF-κB luciferase reporter construct together with 100 ng MAVS plasmid or NF-kB super-repressor were transfected into the cells. The plate was read in a luminometer 24 h post-transfection.
(5.37 MB TIF)

Figure S7 SARS-CoV proteins were expressed in HEK293T cells. Twelve SARS-CoV protein-encoding sequences were cloned into expression vectors with an HA tag. HEK293T cells were seeded in 6-well plate and transfected with 1 µg plasmid of each protein when cells reached 60% confluence, all cells were harvested and lysed 24 h post-transfection for Western blotting analysis with anti-HA antibody.
(7.49 MB TIF)
