# Supplementary figures and images for: MAVS-Mediated Apoptosis and Its Inhibition by Viral Proteins
Source: PLoS One. 2009 Mar 7;4(5):e5466. doi: 10.1371/journal.pone.0005466 (PMC2674933; doi:10.1371/journal.pone.0005466)

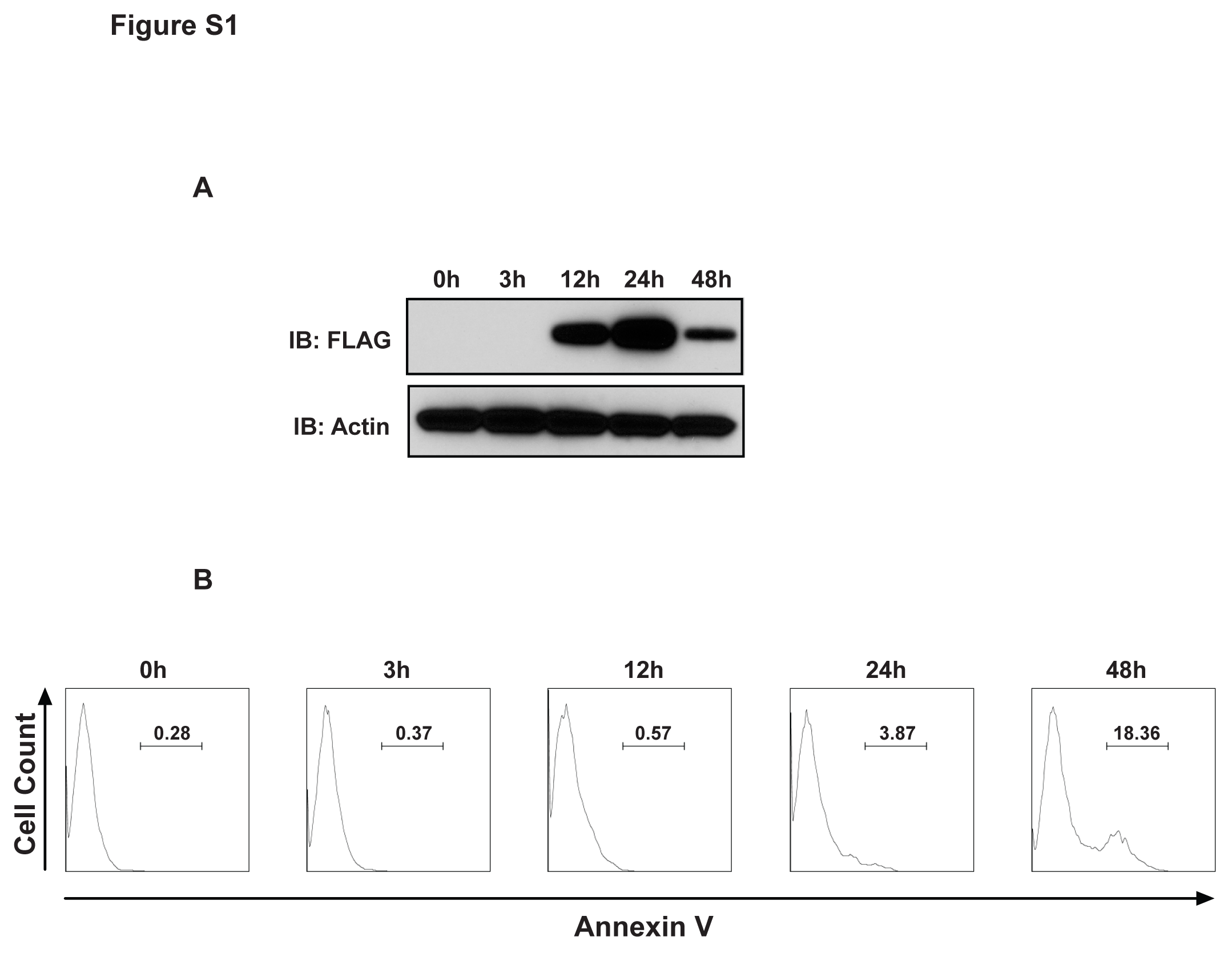

Supplement: Figure S1 — The kinetics of MAVS expression and MAVS-induced apoptosis. (A) 5×10^5^ HEK293T cells were plated in 6 well plate, 3 µg MAVS plasmids were transfected at 3 h, 12 h, 24 h and 48 h prior to cell harvesting. Half of the cells were lysed in RIPA buffer and blotted with anti-FLAG to determine the protein expression kinetics. (B) The other half of the cells from each well were stained with Annexin V and analyzed by flow cytometry. (11.68 MB TIF) [file pone.0005466.s001.tif]

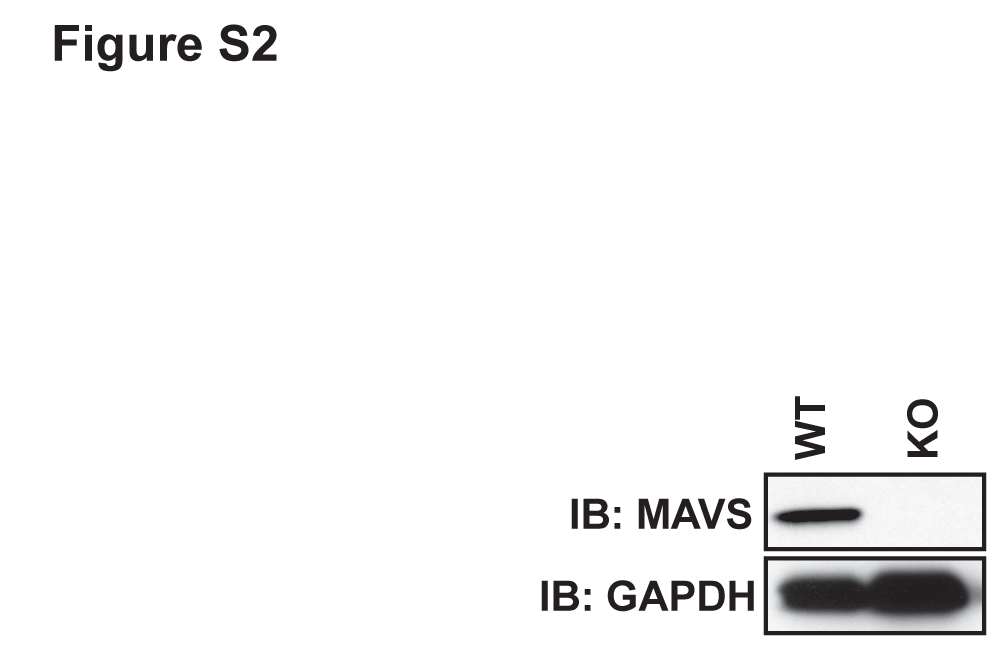

Supplement: Figure S2 — Confirmation of MAVS knockout by Western Blot. One million MAVS+/+ and MAVS−/− MEFs were lysed in RIPA buffer containing proteinase inhibitor cocktail. Cell extracts were subjected to SDS-PAGE and Western blotting for rodent-specific MAVS. (2.15 MB TIF) [file pone.0005466.s002.tif]

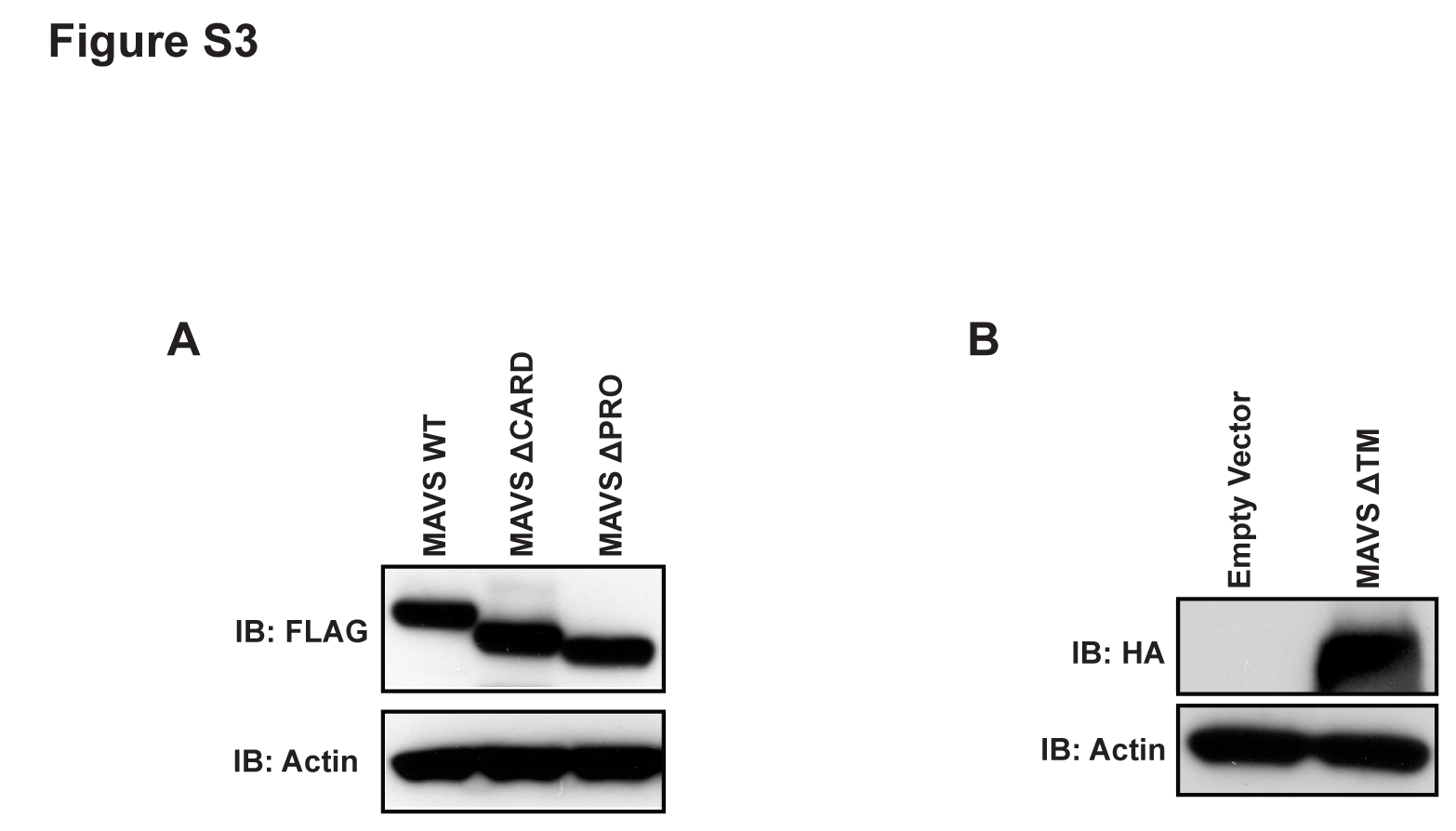

Supplement: Figure S3 — Expression test of MAVS truncation mutants. 3 µg FLAG-MAVS wild type, FLAG-MAVSΔCARD, FLAG-MAVSΔpro and HA-MAVSΔTM plasmids were transfected to 5×10^5^ HEK-293T cells seeded in 6 well plate. Cell were harvested 24 h post-transfection and blotted with anti-FLAG or anti-HA antibody to confirm protein expression efficiency. (4.71 MB TIF) [file pone.0005466.s003.tif]

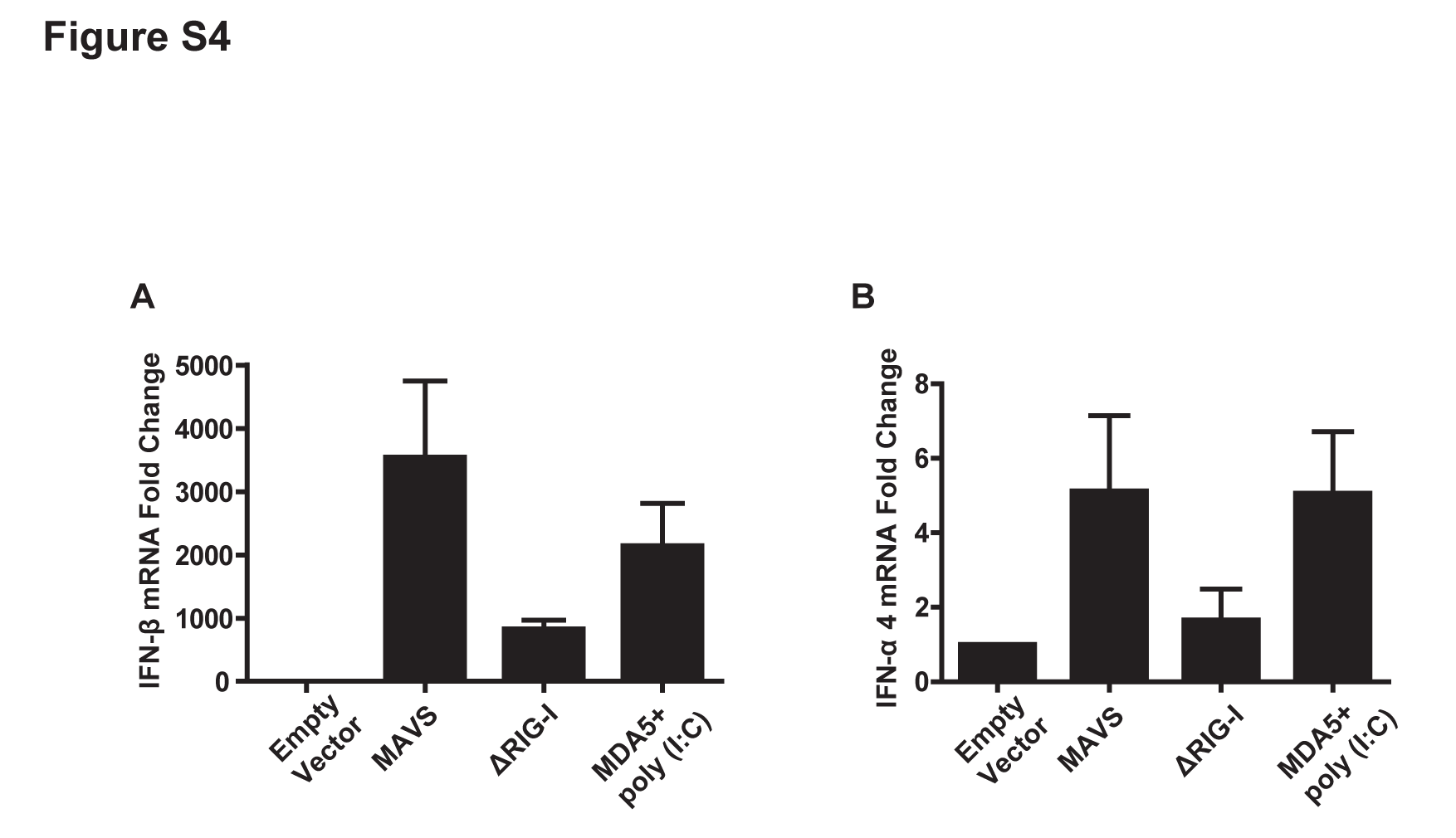

Supplement: Figure S4 — MAVS, ΔRIG-I and MDA5 plus Poly (I∶C) induce increased transcription of IFNB1 and IFNA4. 5×10^5^ HEK293T cells were seeded in 6-well plates and grown overnight. 1 µg of MAVS and ΔRIG-I plasmids were transfected into the cells the next day, similarly 1 µg MDA5 and 100 ng poly (I∶C) were co-transfected into the cells as well. RNA samples were extracted from each group of cells 24 h post-transfection and subjected to real time RT-PCR analyses on the transcripts of IFNB1 and IFNA4. (5.59 MB TIF) [file pone.0005466.s004.tif]

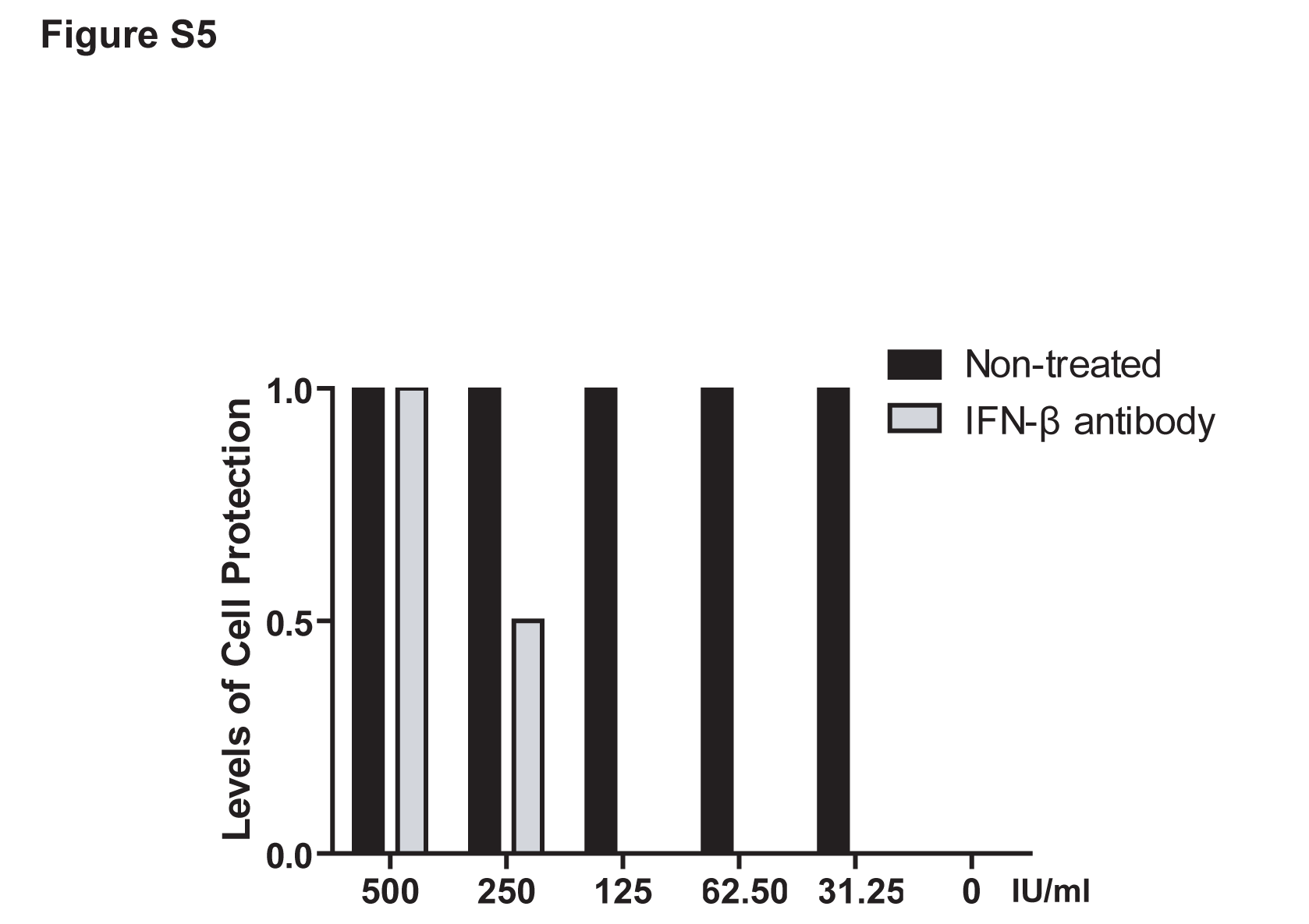

Supplement: Figure S5 — IFN-β neutralizing antibody is able to block the function of secreted IFN-β. A549 cells were seeded in 96-well plate at the density of 2×10^4^ per well, 1 µl of media or anti-IFN-β antibody was added to reach the final concentration of 200 neutralization IU/ml the next day. One hour after incubation with the antibody, recombinant IFN-β was added to culture at a series of doses from 31.25 to 500 IU/ml. Cells were infected with Encephalomyocarditis virus (EMCV) at 4×10^6^ pfu/ml 16 h after IFN treatment. The plates were blind scored 24 h post-infection using “1” as maximal protection (most cells are protected by IFN), “0.5” as about 50% of the cells were not protected by IFN, and “0” as no IFN protection (most cells are dead). (6.29 MB TIF) [file pone.0005466.s005.tif]

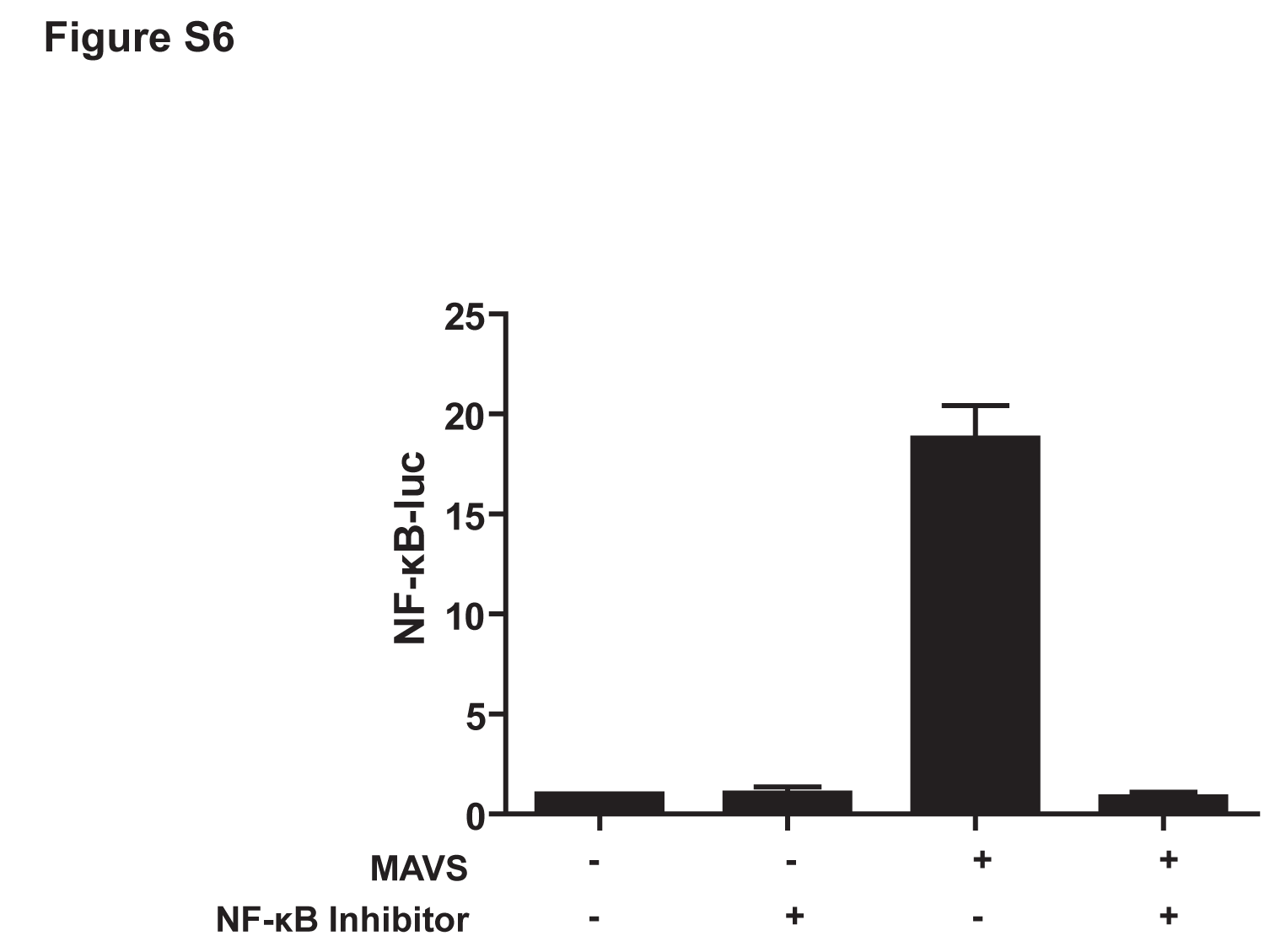

Supplement: Figure S6 — NF-κB super-repressor inhibits the activity of NF-κB. HEK293T cells were seeded in 96-well plate at the density of 1×10^4^ per well and grown overnight. 25 ng of NF-κB luciferase reporter construct together with 100 ng MAVS plasmid or NF-kB super-repressor were transfected into the cells. The plate was read in a luminometer 24 h post-transfection. (5.37 MB TIF) [file pone.0005466.s006.tif]

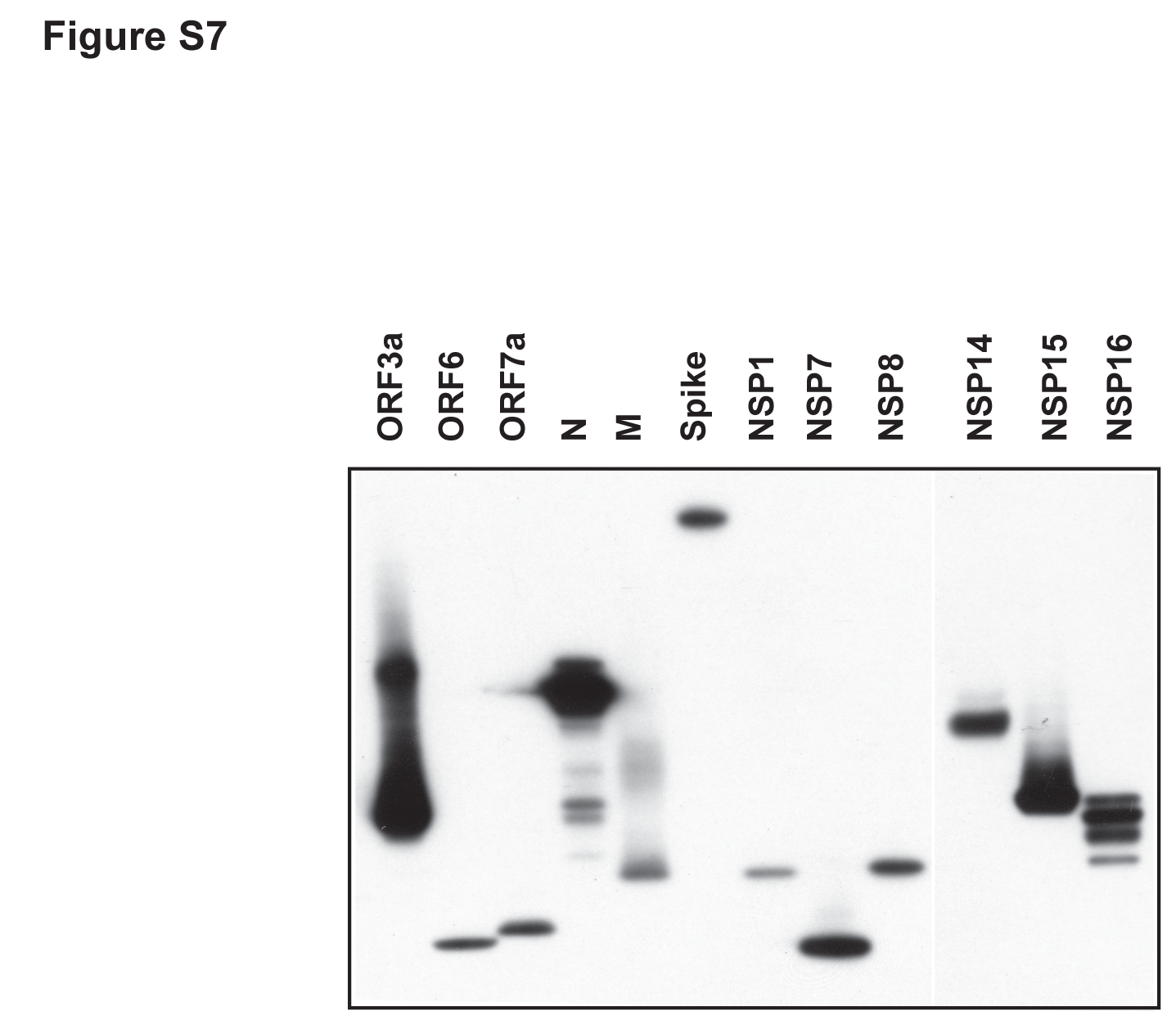

Supplement: Figure S7 — SARS-CoV proteins were expressed in HEK293T cells. Twelve SARS-CoV protein-encoding sequences were cloned into expression vectors with an HA tag. HEK293T cells were seeded in 6-well plate and transfected with 1 µg plasmid of each protein when cells reached 60% confluence, all cells were harvested and lysed 24 h post-transfection for Western blotting analysis with anti-HA antibody. (7.49 MB TIF) [file pone.0005466.s007.tif]
